# Quantum anomalous Hall edge channels survive up to the Curie temperature

**DOI:** 10.1038/s41467-021-25912-w

**Published:** 2021-09-22

**Authors:** Kajetan M. Fijalkowski, Nan Liu, Pankaj Mandal, Steffen Schreyeck, Karl Brunner, Charles Gould, Laurens W. Molenkamp

**Affiliations:** 1 Faculty for Physics and Astronomy (EP3), Universität Würzburg, Am Hubland, D-97074 Würzburg, Germany; 2 Institute for Topological Insulators, Am Hubland, D-97074 Würzburg, Germany

**Keywords:** Electronic properties and materials, Quantum Hall, Topological insulators

## Abstract

Achieving metrological precision of quantum anomalous Hall resistance quantization at zero magnetic field so far remains limited to temperatures of the order of 20 mK, while the Curie temperature in the involved material is as high as 20 K. The reason for this discrepancy remains one of the biggest open questions surrounding the effect, and is the focus of this article. Here we show, through a careful analysis of the non-local voltages on a multi-terminal Corbino geometry, that the chiral edge channels continue to exist without applied magnetic field up to the Curie temperature of bulk ferromagnetism of the magnetic topological insulator, and that thermally activated bulk conductance is responsible for this quantization breakdown. Our results offer important insights on the nature of the topological protection of these edge channels, provide an encouraging sign for potential applications, and establish the multi-terminal Corbino geometry as a powerful tool for the study of edge channel transport in topological materials.

## Abstract (web summary)

The quantum anomalous Hall effect has so far been limited to temperature of the order of 20 mK. Here, Fijalkowski et al. report the existence of chiral edge channels up to the Curie temperature of bulk ferromagnetism of the magnetic topological insulator with a multi-terminal Corbino geometry.

## Introduction

The quantum anomalous Hall effect (QAHE), first discovered in Cr/V-doped (Bi,Sb)_2_Te_3_^1–3^, has opened new avenues for academic studies into solid state manifestations of axion electrodynamics^4–9^ and unconventional magnetism^10–14^. However, the metrological precision of Hall resistance quantization at zero magnetic field so far remains limited to temperature of the order of 20 mK^15–17^, while the Curie temperature (*T*_C_) in the involved materials is as high as 20 K^2,3^.

Given this discrepancy in the temperature scales, a question that has been around since the discovery of the QAHE pertains to the relevant energy scale that stabilizes the protection of the topological state. As the Hall resistance takes on values clearly below *h*/*e*^2^ (where *h* is Planck’s constant and *e* the elementary charge) at temperatures above 1 K or so, well below *T*_C_, where the bulk remains robustly ferromagnetic, the nonquantized Hall resistance can in principle originate from the ordinary bulk states in the absence of any chiral edge channel. Moreover, the possible presence of two distinct ferromagnetic phases^14^ and the otherwise rich magnetic behavior reported at low temperature^10,13,18–21^ leave open the possibility of a second phase transition being involved, and thus possibly playing a role in the edge channel formation at some temperature below *T*_C_.

Recently, experimental progress has been made in significantly increasing the zero magnetic field anomalous Hall resistance at somewhat higher temperatures^22,23^ by modulating or mixing the magnetic dopants. However, these results are still at temperatures well below *T*_C_, and it remains unclear what energy scale drives these observations, as they do not result from a change in the bandgap of the material, nor any other clearly relevant sample characteristic. Other previous reports have suggested that the chiral edge channels coexist with other conducting channels at higher temperatures. This includes investigations of different nonlocal measurement configurations^24^, and nonreciprocal signals^25^, both in a traditional Hall bar geometry. The main challenge for analyzing any results obtained from a traditional Hall bar, is the fact that the signals measured at the voltage leads can result from the current carried by both the edge channels and the bulk states in the material, making it impossible to definitively rule out contribution from the ordinary anomalous Hall effect.

Another established transport geometry is a two-terminal Corbino ring. The ring shape allows for circulating current components, and provides access to additional bulk transport properties^26,27^. In the context of topological materials with edge channels, the obvious limitation for such traditional Corbino setting is the absence of material edges extending directly between both contacts, making such measurement sensitive solely to the conductance in the bulk, and insensitive to the existence of edge channels.

In this work, we propose a solution that combines the two geometries, and opens up the possibility of directly probing the existence of chiral edge channels regardless of bulk conductance in the sample. A key strategy to distinguish between the quantum anomalous Hall effect, and the anomalous Hall effect of the bulk ferromagnet, is to spatially separate the current paths taken by bulk and edge channel transport contributions. Here, we achieve a clear separation of current paths using a non-local measurement scheme in a novel multiterminal Corbino geometry. The measured signals directly reveal that the quantum anomalous Hall edge channels survive up to the *T*_C_ of bulk ferromagnetism in a magnetic topological insulator.

## Results and discussion

### The multiterminal Corbino device

**Fig. 1:**
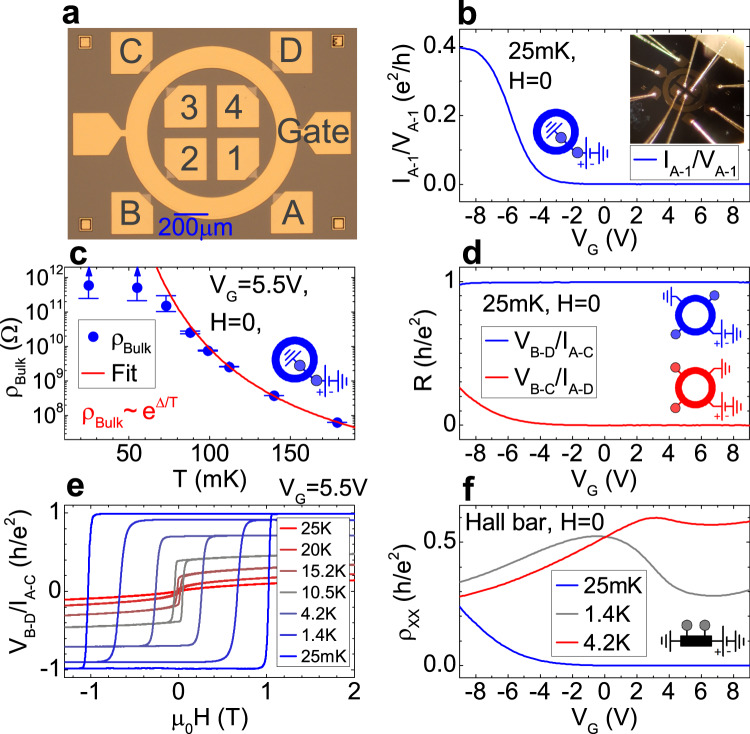
Basic characterization of the multiterminal Corbino device. **a** Optical microscope image of the multiterminal Corbino device with labeled ohmic contacts on the inner edge (1–4), on the outer edge (A–D), as well as a top gate. The smaller image in (**b**) shows a photograph of a mechanically bonded device. **b** A base temperature two-terminal conductance gate sweep measurement between contacts A and 1 (between the two edges). **c** A thermal activation of the bulk resistivity, with the red line representing an Arrhenius fit with an activation energy of 1.11 K. The upper and lower value for the error bars are determined by one standard deviation on the signal measured at the reference resistor. At 25 and 55 mK the upper value is not determined as the standard deviation exceeds the mean value of the signal (schematically indicated by the blue arrows). **d** Four-terminal measurements on the outer edge, at base temperature, showing perfect dissipationless chiral edge channel transport. **e** External magnetic field hysteresis loop of *V*_B-D_/*I*_A-C_ for temperatures ranging from 25 mK to 25 K. **f** Longitudinal resistivity measurements collected from an ordinary Hall bar device (with width of 200 μm and aspect ratio 3:1), patterned from the same MBE layer as the Corbino device.

The multiterminal Corbino device is presented in Fig. 1a. The structure is patterned from a 8.2 nm thick V_0.1_(Bi_0.2_Sb_0.8_)_1.9_Te_3_ layer grown by molecular beam epitaxy (MBE)^28^, and optimized for exact anomalous Hall resistance quantization^15^. The outer diameter of the ring is 1 mm, the width between the edges is 100 μm, and the width of each contact is 15 μm. Four AuGe ohmic contacts along each outer and the inner edge allow for four-terminal measurements along the same edge in the low temperature perfectly quantized state, and also for various measurement configurations involving the two edges. These allow a clear determination of the role of edge channels. The sample is also fitted with an AlOx/HfOx/Au top gate for tuning of the Fermi level. The width of 100 μm between the edges is large enough to clearly decouple the chiral edge channels residing on both edges, when the bulk is insulating. This is empirically verified by measurements demonstrating perfect quantization at low temperature (see Fig. 1).

### Basic characterization

We first consider a two-terminal measurement applying a voltage between contact A and contact 1 (which is grounded), and measuring the flowing current with a series resistor. Such data are presented in Fig. 1b at a temperature of 25 mK as a function of gate voltage. For gate bias values in the QAHE plateau region (with the center of the plateau at about +5.5 V), the current, and thus the conductance vanishes. Indeed, below 55 mK the two-terminal resistance *V*_A-1_/*I*_A-1_ is noise limited (by the noise on the voltage drop over a series reference resistor) with a lower bound estimate of 11.2 GΩ. Given the high conductance of edge channels running along each edge, in comparison to the bulk conductance at such low temperatures, equipotential rings should occur on the outer and the inner edges of the Corbino. This is confirmed experimentally by measuring the potential at the remaining contacts, which for each edge are all equal to each other to well within the 1% experimental uncertainty on the lock-in amplifiers. For the purposes of determining the bulk resistivity, we effectively then have a Corbino with one inner and one outer contact, and the aspect ratio entering the calculation is given by the width of the ring divided by its circumference. This ratio is approximately 1/28, yielding a lower bound of 310 GΩ per square for the bulk sheet resistivity (*ρ*_Bulk_). That such an extremely high resistivity can be measured in a cryostat is a first example of the usefulness of the multi-terminal Corbino geometry. The Fig. 1c shows a thermal activation plot of bulk resistivity with an Arrhenius activation energy corresponding to a temperature (Δ in the Fig. 1c) of 1.11 K.

When four-terminal measurements are performed at 25 mK along a single edge, perfectly dissipationless chiral edge channel transport is observed. In Fig. 1d we plot the *V*_B–D_/*I*_A–C_ (blue color) and the *V*_B–C_/*I*_A–D_ (red color) resistances, revealing similar characteristics to *R*_xy_ and *R*_xx_ of a perfectly quantized state in a traditional Hall bar, where *V*_B–D_/*I*_A–C_ reaches a quantized value of *h*/*e*^2^ and *V*_B–C_/*I*_A–D_ vanishes.

In Fig. 1e we show a magnetic field sweep of the *V*_B–D_/*I*_A–C_ signal for temperatures from 25 mK to 25 K. This configuration corresponds to an *R*_xy_ in a traditional Hall bar. At 25 mK (blue color) the curve has a rectangular-shaped hysteresis loop and shows perfect quantization. When the temperature is increased the signal decreases, and at temperatures around 18 K, the Curie temperature of the film, the zero magnetic field Hall signal vanishes.

Figure 1f shows longitudinal resistivity *ρ*_xx_ obtained from a Hall bar patterned from the same MBE layer. The *ρ*_xx_ value measured here results from the combined effect of the bulk resistivity and the edge channel contribution. This can lead to a non-monotonicity as seen most clearly at 1.4 K, which reflects the competing effects of bulk resistivity increasing as the Fermi level enters the bulk gap, and the dissipationless edge channel shorting the two voltage contacts when backscattering through the bulk is suppressed.

### Nonlocal measurements

To gain insight on the role of edge channels as a function of temperature, we turn to nonlocal measurements, involving both edges of the Corbino. Specifically, we apply a bias voltage to contact A with contact 1 grounded, and record the individual potentials (with reference to the ground) at the remaining contacts, as a function of gate voltage and temperature.

**Fig. 2:**
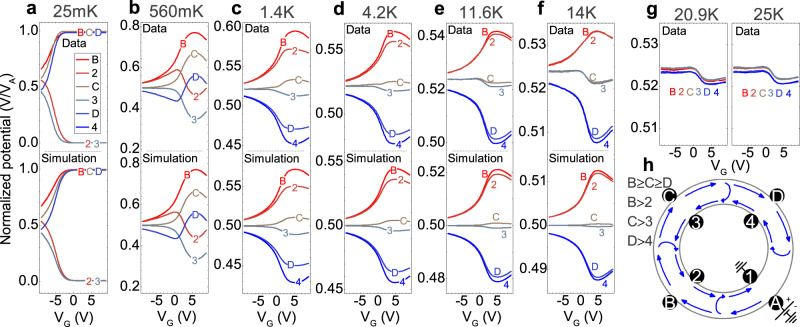
Nonlocal measurements. Individual contact potentials in the magnetized state at zero external magnetic field, normalized to the bias voltage. The bias voltage is applied to contact A with contact 1 grounded. The temperature of the measurement is: 25 mK (**a**), 560 mK (**b**), 1.4 K (**c**), 4.2 K (**d**), 11.6 K (**e**), 14 K (**f**), and 20.9 K with 25 K (**g**). Below the data in **a**–**f**, the corresponding simulated curves are plotted. (Contact 4 was not accessible during the measurement run below 1.4 K). At 1.4 K and above, the measurements of contacts B and 2 were multiplied by a factor of 1.00357, and contacts C and 3 by a factor of 0.9943, to correct for the amplifier gain differences between the different instruments in the experiment. Color coding is the same in (**a**–**g**). **h** A schematic of the system’s chirality, with a description of the contact voltage order stemming from the chiral edge channel transport with bulk dissipation.

In the top half of each panel in Fig. 2, we plot zero magnetic field gate voltage sweeps for this configuration, at various temperatures. In all cases, the potentials are normalized to the voltage applied at contact A (typically 100 μV below 1 K, and 5 mV at higher temperatures). In the low-temperature regime, when the system is tuned into the QAHE plateau (around +5 V), we observe that contacts along the outer edge are equipotential to the biased contact while those along the inner edge are equipotential to ground, consistent with pure chiral edge channel transport and a fully insulating bulk.

Richer behavior is observed when bulk conductivity is turned on, either by tuning the Fermi level with gate voltage or by increasing the temperature. For all temperatures, including 25 mK where perfect quantization is observed, at a gate voltage of −9V, the Fermi level is far away from the charge neutrality point, and the bulk is thus conducting. As part of the current can now directly flow from contact A to 1, the equipotential situation along the outer and inner edges vanishes, and the potentials at the remaining contacts take on values closer to 0.5, the average potential between A and 1. Noteworthy, however, is that a clear chirality is observed in the data with the potential *V*_B_ > *V*_C_ > *V*_D_ and *V*_2_ > *V*_3_ > *V*_4_ for all temperatures. This shows that even though the bulk is conducting, chiral edge channel transport remains readily accessible.

We now examine a gate voltage of +5 V, where the Fermi-level is in the bulk gap, and pure edge channel transport is observed at low temperatures. As the temperature is increased, the conductivity of the bulk is progressively turned on. This gradually changes the ratio of edge channel to bulk conductivity, and causes the potential at the detection contacts to move towards the average value of 0.5 (note that for better visibility of the data, the y axis changes between the various temperatures in Fig. 2). Also in this case, the above described chirality is observed.

The situation only changes in panel 2g, which shows the behavior of the potentials above the 18 K Curie temperature of the material. Here all contacts are, to within experimental error, equipotential to each other. The value of 0.52 instead of 0.5 results from a slight difference in contact resistance between contacts A and 1, and the change in this value around 0 V gate voltage comes from the effect of the gate on the resistance of these contacts.

We qualitatively interpret these data as follows: In the absence of any edge channels, one would expect a fairly direct potential gradient from contacts A to 1. This highlights the key difference from a typical Hall bar geometry, which allows us to rule out any bulk anomalous Hall contribution to the observed voltages. The origin of the anomalous Hall effect of a bulk ferromagnet is completely distinct from the quantum anomalous Hall effect. It stems from the intrinsic and extrinsic mechanisms resulting in asymmetric scattering of current-carrying electrons, and thus only plays a role where current is flowing. Since, in contrast to the case of a Hall bar, no bulk current flows near the voltage leads in the Corbino geometry, the anomalous Hall effect has no effect on our measurements. The potentials resulting from bulk transport, measured at the positions of all voltage contacts, are therefore very close to 0.5, and in particular *V*_B_ = *V*_D_ (*V*_2_ = *V*_4_) due to symmetry.

The presence of chiral quantum anomalous Hall edge channels leads to an actual current flow as depicted in Fig. 2h. Current exiting contact A moves clockwise (for the given magnetization) around the outer edge, and is also able to partly backscatter through the bulk to the inner edge channel where it moves counter-clockwise. This produces both the observed chirality *V*_B_ > *V*_C_ > *V*_D_, as well as the finite voltage drops between the outer and inner edge at all points around the ring. Of course, the opposite chirality is observed for reversed magnetization. Above 18 K, the QAHE edge channels disappear, and we recover the case corresponding to only a conducting bulk.

### Landauer–Büttiker modelling

**Fig. 3:**
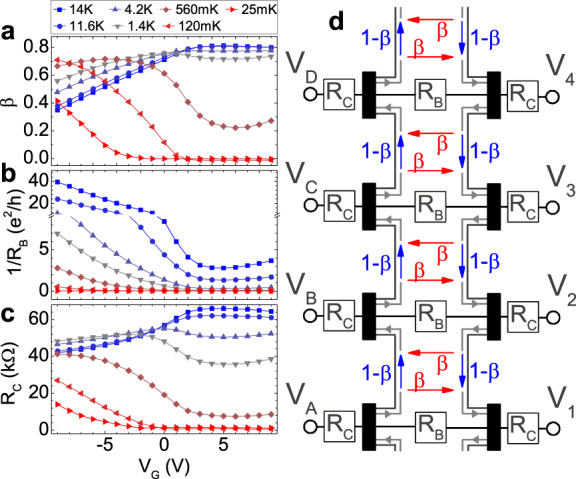
Landauer-Büttiker parameters. Gate voltage and temperature evolution of the fitting parameters (see description in the text): **a** *β*, **b** 1/*R*_B_, and **c** *R*_C_. **d** Schematic of the device layout describing the model. The red and blue arrows represent the transmission coefficients dependent on *β*. Resistors *R*_C_ simulate the lead resistance in series with every contact, and resistance *R*_B_ the inverse bulk conductance.

To further quantify our interpretation, we use a simple model based on the Landauer–Büttiker formalism^29^. The model is depicted in the schematic in Fig. 3d and consists of an eight-contact Landauer–Büttiker network to describe the chiral edge channels. The role of the conducting bulk is accounted for in two ways: by a set of resistors *R*_B_ linking each pair of inner and outer contacts to account for bulk conductivity (treated in the formalism as an additional transmission channel), and by a scattering parameter *β* that allows for backscattering between the outer and the inner edge channels at ring positions between the contacts. Given the much larger distances involved, bulk conductivity between non-neighboring contacts can be safely neglected. This network leads to eight linear equations with two free parameters (*β* and *R*_B_) that can be solved analytically for any combination of current and voltage contacts.

The final element needed to completely describe the data is *R*_C_, which is the contact/lead resistance at each of the eight ohmic contacts. This *R*_C_ primarily stems from the sheet resistance of the mesa constriction between the metallic contact and the ring. This minimalistic model with only three adjustable fitting parameters is sufficient to describe all measurements. These three parameters can be jointly obtained by fitting the model to the following three measurements: The two-terminal resistance *V*_A-1_/*I*_A-1_, the two-terminal resistance *V*_A–C_/*I*_A–C_, and the four-terminal (effective *R*_xy_) resistance *V*_B–D_/*I*_A–C_ (see the Methods section for more details). Once the three parameters are obtained, they are used to generate the simulations shown in the bottom panels of Fig. 2, for our main measurement configuration. We emphasize that no fitting to the data in Fig. 2 was performed, as the model is already fully determined by the three above listed measurements for any given temperature and gate voltage.

The actual parameters used to generate the simulations in Fig. 2 are given in Fig. 3a–c as a function of gate voltage and temperature. The dependence of the contact/lead resistance *R*_C_ of Fig. 3c on both temperature and gate voltage matches that of the total mesa resistivity of the material as obtained from measurements on a Hall bar (shown in Fig. 1f). Note that this resistance results from the parallel conductance of the bulk and an edge channel, and is thus distinct from the pure bulk resistance *R*_B_ of Fig. 3b. The inverse bulk resistance (1/*R*_B_) also follows the expected behavior, with conductivity turning on with either temperature or as the gate voltage is tuned away from the plateau regime. The dependence of the parameter *β* (Fig. 3a) roughly correlates with the bulk conductivity, consistent with the bulk mediating backscattering between the two edges. The somewhat nonmonotonic behavior may result from the fact that when the bulk gets highly conducting, backscattered carriers begin to find pure bulk paths to the other contacts, which is not accounted for in our basic model. Note that a value of *β* = 0 represents ideal edge channel conduction with a fully insulating bulk, whereas *β* = 1 would be tantamount to a complete destruction of the edge channel. Values in between are a measure of the relative weight of bulk vs. edge channel conduction.

Note that the existence of a contact resistance *R*_C_ also limits the degree of chiral splitting observable in the experimental data of Fig. 2. For example, the spread from 0.53 to 0.51 between contacts B and D at 14 K. The asymmetry between B and D produced from the Corbino contribution itself, is thus larger than the 2% seen in the figure. This was confirmed with measurements on a second sample (Supplementary Note 1 and Supplementary Fig. 1) with wider leads (50 μm) to minimize the contact resistance. It has a splitting of approx. 8% under comparable conditions.

**Fig. 4:**
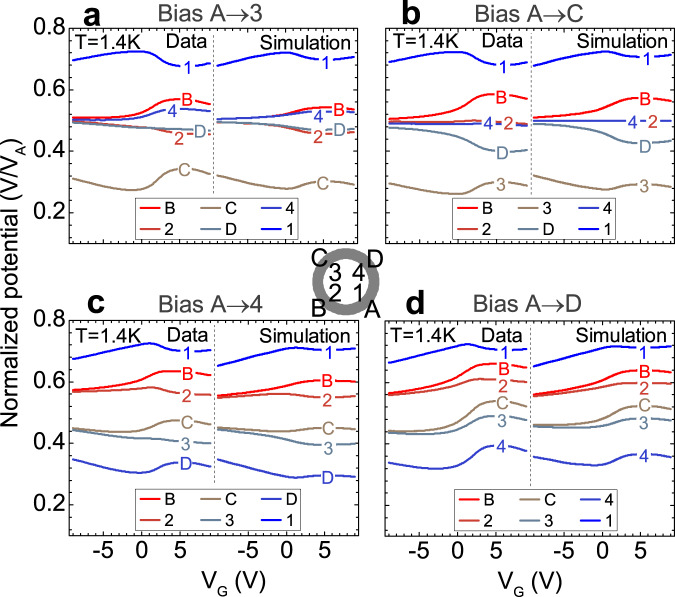
Various bias configurations. Side by side comparison of the experimentally measured individual potentials in the magnetized state at zero external magnetic field (left in each figure), and simulated using the model (right in each figure), normalized to the bias voltage. All curves were collected at 1.4 K. Biased contact pairs (source-drain) in the figures are: A-3 (**a**), A–C (**b**), A-4 (**c**), and A–D (**d**).

The robustness of our model can be further tested by comparing it to additional measurement configurations, again without introducing any new fitting parameters. A sampling of other source-drain contact arrangements is presented in Fig. 4. In all cases, good agreement between the model and the data confirms the existence of chiral edge channel transport.

Our observations thus clearly show that the quantum anomalous Hall chiral edge channels survive over a temperature range vastly exceeding the regime where quantization is directly observable in the experiment. This implies that the breakdown mechanism for this quantization is not a feature of the topological edge channels, but rather results from parasitic conductance through a nonfully insulating bulk. This in turn suggests that the topological protection in these systems is robust against temperature, and that efforts to increase operation temperatures for potential future applications should focus on optimizing the insulating properties of the bulk. In addition, these results establish the multi-terminal Corbino geometry as a powerful experimental tool for studies of edge channel transport in topological materials.

## Methods

### Device preparation

Our magnetic topological insulator V_0.1_(Bi_0.2_Sb_0.8_)_1.9_Te_3_ layer is grown using molecular beam epitaxy (MBE) on a Si(111) substrate^28^. The layer is capped in-situ with an 8 nm thick insulating Te layer as a protection from ambient conditions, as well as from chemicals in the lithographic process. The magnetic TI layer thickness is determined using X-ray reflectivity (XRR) measurements to be approximately 8.2 nm. A multiterminal Corbino geometry is patterned using standard optical lithography methods, with AuGe ohmic contacts and an AlOx/HfOx/Au top gating layer stack allowing for tuning of the Fermi level.

### Transport measurements

Transport measurements are performed in a ^3^He-^4^He dilution refrigerator system (for measurements below 1 K) and in a ^4^He cryostat (for measurements above 1 K), with an out-of-plane external magnetic field. Measurements are done in the linear response regime using low-frequency AC voltage excitation (below 20 Hz), with the exception of the bulk resistivity plotted in Fig. 1c where a quasi-DC square wave (approx. 4 mHz) excitation is used instead.

### Method of obtaining the model parameters

The three parameters (*β*, *R*_B_, *R*_C_) can be obtained by matching the three experimentally measured resistances (*R*_A1,A1_ = *V*_A-1_/*I*_A-1_, *R*_AC,AC_ = *V*_A–C_/*I*_A–C_, *R*_BD,AC_ = *V*_B–D_/*I*_A–C_) to the same resistance configurations calculated from the Landauer–Büttiker network. By analytically solving the Landauer–Büttiker circuit for these configurations of current and voltage leads, one obtains the following equations:

1 $${R}_{{{{{{{{\rm{A1,A1}}}}}}}}}=\frac{h}{{e}^{2}}\frac{3\beta +1+4/{R}_{{{{{{{{\rm{B}}}}}}}}}}{4(\beta +1/{R}_{{{{{{{{\rm{B}}}}}}}}})(1+1/{R}_{{{{{{{{\rm{B}}}}}}}}})}+2{R}_{{{{{{{{\rm{C}}}}}}}}}$$

2 $${R}_{{{{{{{{\rm{AC,AC}}}}}}}}}=\frac{h}{{e}^{2}}\frac{1}{1-\beta }+2{R}_{{{{{{{{\rm{C}}}}}}}}}$$

3 $${R}_{{{{{{{{\rm{BD,AC}}}}}}}}}=\frac{h}{{e}^{2}}\frac{1}{1+1/{R}_{{{{{{{{\rm{B}}}}}}}}}}$$

which form a system of independent equations relating the three model parameters (*β*, *R*_B_, *R*_C_) to the three measured resistances (*R*_A1,A1_, *R*_AC,AC_, *R*_BD,AC_).

## Supplementary information

Supplementary Information

## Data Availability

All data necessary to support the conclusions of the paper are available in the manuscript. Source data are provided with this paper.

## Source data

Source Data
