# Supplementary material for: Quantum anomalous Hall edge channels survive up to the Curie temperature
Source: Nat Commun. 2021 Sep 22;12:5599. doi: 10.1038/s41467-021-25912-w (PMC8458438; doi:10.1038/s41467-021-25912-w)
Supplement: Supplementary file 1 — Supplementary Information [file 41467_2021_25912_MOESM1_ESM.pdf]

Quantum anomalous Hall edge channels survive up to the Curie temperature - Supplementary material

Kajetan M. Fijalkowski,[1,2] Nan Liu,[1,2] Pankaj Mandal,[1,2] Steffen Schreyeck,[1,2]
Karl Brunner,[1,2] Charles Gould,[1,2] and Laurens W. Molenkamp[1,2]
[1]*Faculty for Physics and Astronomy (EP3), Universität Würzburg, Am Hubland, D-97074, Würzburg, Germany*
[2]*Institute for Topological Insulators, Am Hubland, D-97074, Würzburg, Germany*
(Dated: August 31, 2021)

SUPPLEMENTARY NOTE 1 - NON-LOCAL MEASUREMENTS ON A MULTI-TERMINAL CORBINO DEVICE WITH WIDER CONTACTS.

Supplementary Fig. 1 shows non-local measurements collected from a second device, where the contact width is increased from 15 μm (for the sample from the main text) to 50 μm. A bias voltage of 5 mV is applied to contact A, while contact 1 is grounded, and voltages on all of the remaining contacts are measured with reference to the ground. All measured potentials are normalized to the voltage measured at contact A. The potentials are measured as a function of the applied gate voltage, at zero external magnetic field in a magnetized state, at temperatures: 1.4 K (a), 4.2 K (b), 11.6 K (c), 15.2 K (d), and above the Curie temperature, at 20 K (e).

The model parameters for this sample are obtained in the identical way as described in the main text. The corresponding simulated curves are presented in the bottom panels in (a)-(e), and good agreement is found between the data and the simulation. The evolution of the extracted model parameters with gate voltage and temperature is presented in (f). The behavior of the inter-edge scattering β and the bulk conductance $1/R_\mathrm{B}$ is consistent between both samples, and the contact resistance R_C is clearly reduced for the sample with wider contacts. This is consistent with all of the analysis, as the only difference in the device geometry between the two samples is the contact width [see an optical microscope image in (g) for the sample with wider contacts]. The smaller contact resistance leads to a significant increase in the magnitude of the non-local signals, with voltage splitting between contacts B and D of about 8 % at 15.2 K, right below the Curie temperature (which is approx. 18 K).

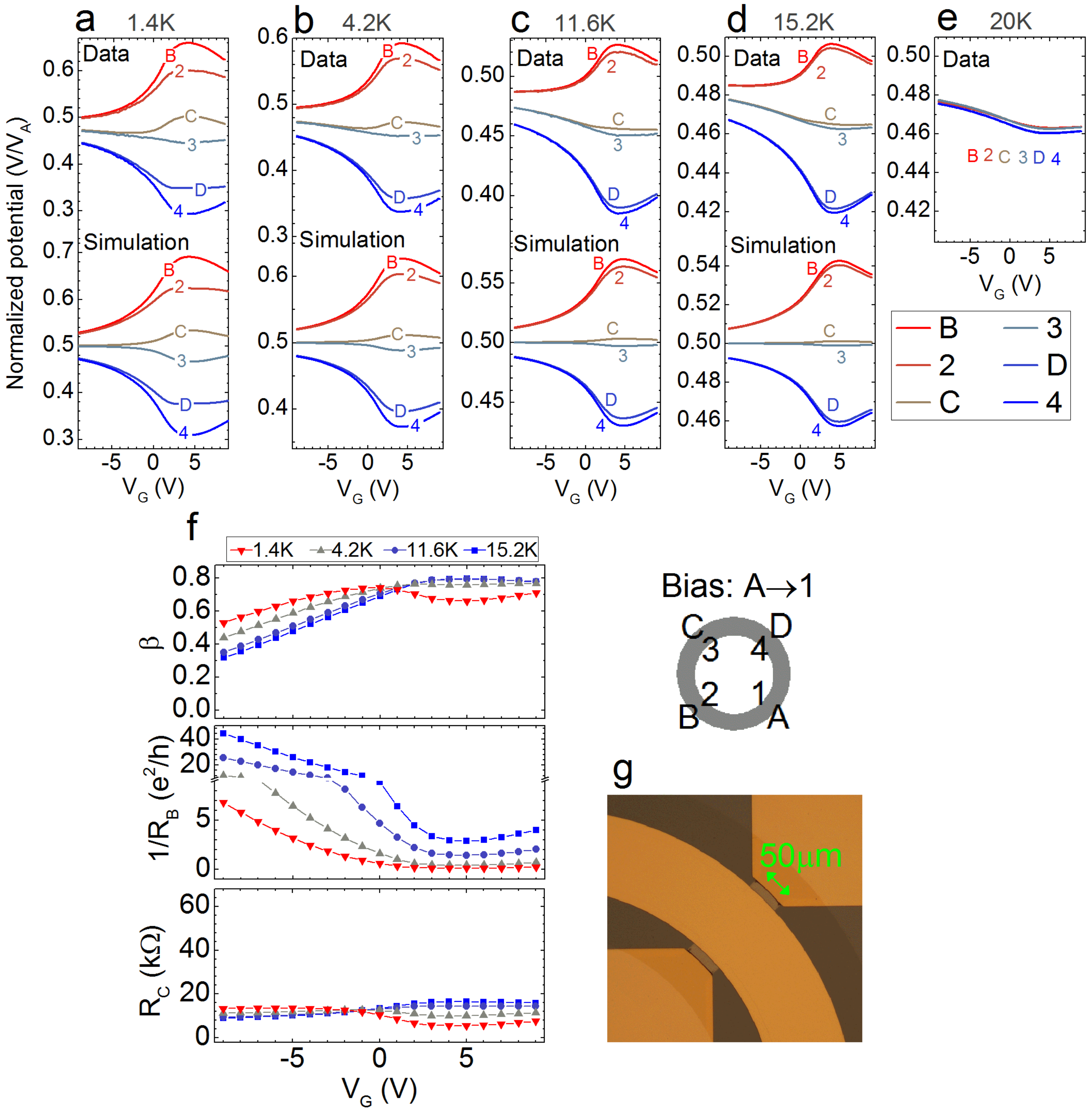

Supplementary Fig. 1. Individual contact potentials collected in the magnetized state at zero magnetic field, from a second sample with wider contacts (50 μm) at a variety of temperatures. Contact A is biased, and contact 1 is grounded. All potentials are normalized to the bias voltage. The measurements of contacts B and 2 were multiplied by a factor of 1.00357, and contacts C and 3 by a factor of 0.9943, to correct for the amplifier gain differences between the different instruments used in the measurements. Color coding is the same in (a-e). f) Corresponding modeling parameters obtained for this sample, using the same methodology as in the paper, as a function of gate voltage. g) A microscope image showing the widened contacts.
